# Self-inflicted tourniquet application resulted with two fasciotomies: Case report of an initially omitted Munchausen case

**DOI:** 10.1016/j.ijscr.2024.109746

**Published:** 2024-05-08

**Authors:** Arman Vahabi, Ali Engin Daştan, Javad Mirzazada, Yener Yoğun, Okan Tezgel, Kemal Aktuğlu

**Affiliations:** a Ege University School of Medicine, Department of Orthopedics and Traumatology, Izmir, Turkey; b VM Medical Park Hospital, Department of Orthopedics and Traumatology, Kocaeli, Turkey; c Ankara University School of Medicine, Department of Orthopedics and Traumatology, Ankara, Turkey; d Van Educational and Research Hospital, Department of Orthopedics and Traumatology, Van, Turkey

**Keywords:** Self-harm, Self-mutilation, Compartment syndrome

## Abstract

**Introduction:**

Compartment syndrome is an emergency which requires prompt intervention. While main challenge typically revolves around determining necessity for fasciotomy in suspected cases, etiology is often pronounced, leaving little room for differential diagnosis.

**Case report:**

We report a case with unconventional presentation and clinical course, ultimately diagnosed as Munchausen Syndrome.

**Discussion:**

It has been reported that individuals with Munchausen syndrome are successful at manipulating healthcare professionals. They often study the symptoms of their sickness, examination findings, and findings that may alert doctors, mastering their techniques over time.

**Conclusion:**

It is of importance to consider Munchausen Syndrome as a potential cause, particularly in cases where clinical history and course of symptoms do not align with our experiences and cannot be reconciled with other possible diagnostic patterns.

## Highlights

• Munchausen syndrome is characterized by patients feigning illness to receive medical treatment without a clear secondary gain.
• While Munchausen syndrome is classified as a psychiatric diagnosis, individuals with this condition often seek medical attention at different clinics.
• Individuals with Munchausen syndrome are successful at manipulating healthcare professionals.

## 1 Introduction

Compartment syndrome in hand and forearm manifests with pain, swelling, numbness, and tingling in its early stages due to increased pressure within anatomical compartments. In advanced stages, circulatory and nerve damage become evident [1]. While instruments exist to measure intracompartmental pressure, diagnosis primarily relies on clinical suspicion [2]. In most cases, clinical history is pronounced with clear history of high energy or repetitive traumas. Delayed or missed diagnosis can lead to permanent nerve or muscle damage, or even necessitate amputation. Therefore, suspected compartment syndrome warrants immediate fasciotomy to alleviate compartment pressures to avoid further damages on viable structures [3].

Munchausen syndrome is a well-established clinical diagnosis characterized by patients feigning illness to receive medical treatment without a clear secondary gain [4]. The primary motivation is typically to garner attention. This syndrome encompasses a broad spectrum of behaviors, ranging from claiming physical symptoms such as chest pain and headache to actively inducing serious symptoms [[5], [6], [7]]. While factors like childhood trauma, serious childhood illness, or personality disorders are considered predisposing factors, the syndrome's etiology remains incompletely understood [8,9]. Despite being recognized for over 70 years; the literature still primarily consists of case reports and reviews.

## 2 Case report

**Fig. 1:**
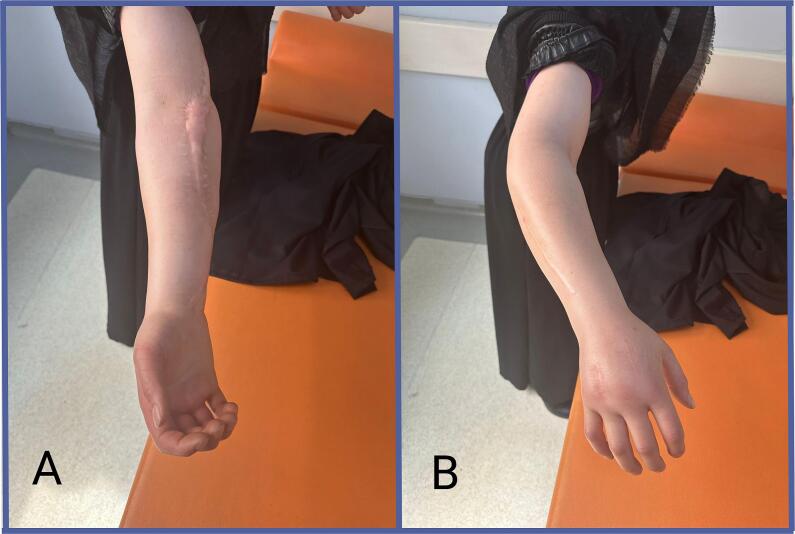
Admission to outpatient clinic after on-going symptoms for 4-days, started with handling a bucket. 2A: Volar aspect of hand and forearm 2B: Dorsal aspect of hand and forearm. Note the swelling in dorsal side, prominent in hand.

This case report is presented in line with SCARE guidelines [10]. 26-years-old female without any comorbidities from rural area presented to outpatient clinic complaining of swelling in the right hand and forearm, accompanied by pain. She reported that the symptoms began four days prior, after lifting a bucket full of water, with a gradual increase in pain and swelling leading up to her visit. Additionally, she mentioned experiencing progressive edema in his arm following a minor trauma, two years prior, which had led to surgical intervention at that time. Previous hospitalization and operation records indicated that fasciotomy had been performed based on a preliminary diagnosis of compartment syndrome. However, the underlying etiology remained unclear despite investigations conducted at that time. On examination, diffuse edema, particularly notable on the dorsal aspect of hand and forearm, and widespread tenderness were noted. Radial and ulnar pulses were palpable, and there were no signs of paresthesia or hypoesthesia. Notably, fasciotomy scars were evident on the dorsal and volar aspect of forearm and hand (Fig. 1). X-rays and upper extremity venous-arterial Doppler ultrasound examinations revealed no abnormalities. The patient was subsequently admitted to the hospital for further observation.

**Fig. 2:**
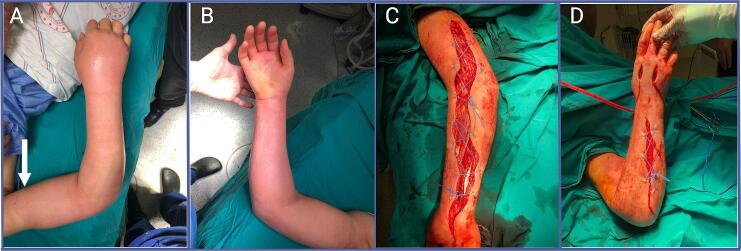
Photographic documentation before and after first fasciotomy. 2A: Dorsal aspect of hand and forearm. Note the levelling in swelling that later we attributed to mark by tourniquet application 2B: Volar aspect of hand and forearm. 2C: Volar aspect of hand and forearm, after first fasciectomy. 2D: Dorsal aspect of hand and forearm, after first fasciotomy.

The patient was initially managed with eau de goulard soaked dressing and elevation. However, despite these measures, after a minor regression following admittance, there was a progression of swelling, particularly at the dorsum of the hand, observed during the morning visit the following day. Patient reported pain with passive stretching and began to experience paresthesia in the hand. Given the suspicion of compartment syndrome, a decision was made to proceed with fasciotomy. However, the underlying cause for the clinical deterioration remained elusive at this point. In an effort to fully elicit the situation, the physician who had performed the previous operation was contacted. He stated that every diagnostic tests conducted during the previous operation yielded negative results, and no underlying cause for compartment syndrome had been identified. Photographic documentation of this incident was subsequently provided (Fig. 2). He also noted that despite the fasciotomy, the patient's postoperative pain persisted until third day post-fasciotomy, until an instance with a nurse. The patient was seen squeezing her arm with a piece of cloth and when questioned about this, she stated that she believed it would alleviate her pain. Our colleague did not provide further elaboration at that time and did not suspect of self-harm since he thought it might be traditional behavior in rural areas.

**Fig. 3:**
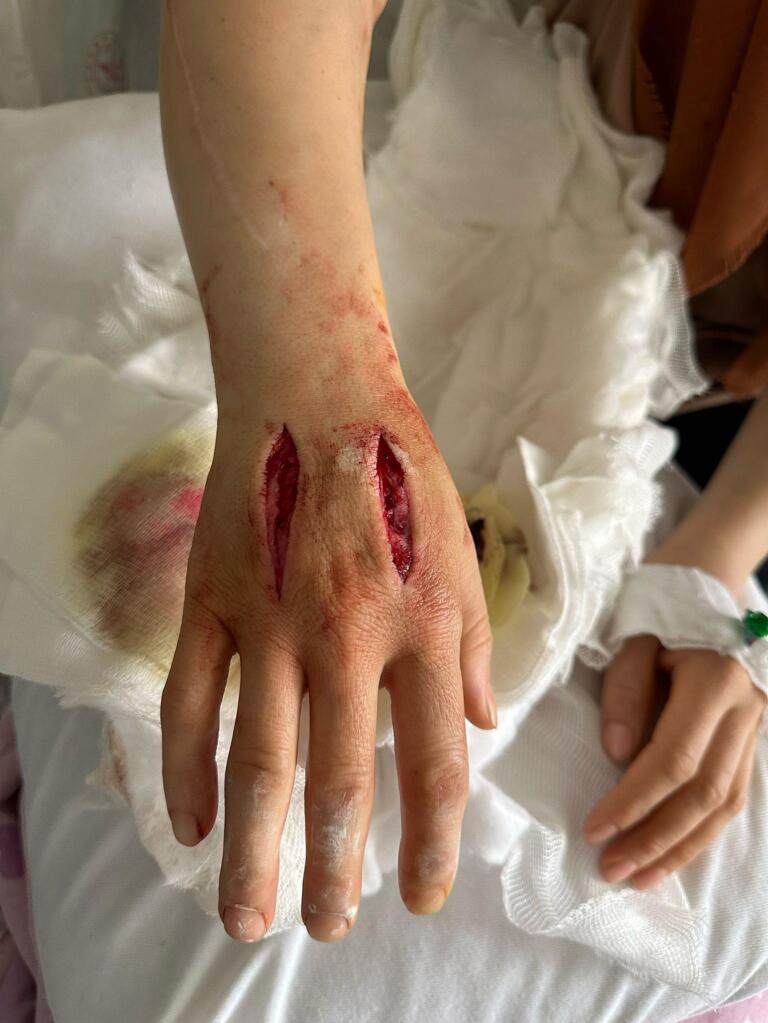
Rapid resolution after 12-hour postoperatively. Note that all swelling is dissolved, and patient is complaint free other than pain at incision sites.

Based on progression of swelling and clinical deterioration despite the absence of an identifiable underlying cause and considering the new information on her previous intervention, we initiated an assessment into the possibility of self-mutilation. Upon raising concerns on issue with her relatives, they expressed that they would not consider this possibility as implausible, and they did not seem surprised. To proceed cautiously, we opted for a less extensive fasciotomy, deferring a more comprehensive psychiatric evaluation until after the surgery. The fasciotomy was performed utilizing the existing incision scars on the dorsum of the hand, with no intervention in the forearm. Remarkably, there was a rapid alleviation of swelling and pain following the intervention (Fig. 3). Subsequently, the fasciotomy incision was closed on the third day post-fasciotomy. Following psychiatric consultations involving the patient and her family, it was decided to initiate psychiatric follow-up, suspecting Munchausen syndrome.

Upon reflection, we came to realize that the significant levelling in swelling and a mark that can be contributed to some kind of tourniquet (Fig. 2A) prior to initial surgery, coupled with her notably indifferent and obedient attitude towards treatment, warranted closer attention, which unfortunately we failed to provide initially. With a thorough evaluation right from the start, with more careful observation and detailed clinical history, this case might have been spared from undergoing four surgeries.

## 3 Discussion

While Munchausen syndrome is classified as a psychiatric diagnosis, individuals with this condition often seek medical attention at different clinics [11,12]. Therefore, it is crucial for all healthcare professionals to be vigilant about recognizing its signs. Diagnosis starts with suspicion and in many instances, including our case, the diagnosis is made based on suspicion and the identification of inconsistencies and mismatching pieces in the patient's medical records and provided history [13]. However, confirming the diagnosis or providing an effective treatment can be challenging, as individuals with Munchausen syndrome often do not perceive themselves as having a problem and may not adhere to treatment recommendations [14,15]. Some approaches advocate directly confronting the patient about their behavior, while others suggest broaching the topic indirectly and referring them to psychiatry. In our case, we consulted the patient's family when suspicion arose. However, to maintain trust in the doctor-patient relationship, we opted not to directly confront with the patient and instead involved a psychiatry consultation in the process.

It has been reported that individuals with Munchausen syndrome are successful at manipulating healthcare professionals [12,16]. They often study the symptoms of their sickness, examination findings, and findings that may alert doctors, mastering their manipulation techniques over time. The absence of an obvious level mark for tourniquet application in our patient's second application, as compared to the photographs taken during her initial visit, may indicate that she became more cautious after being caught red handed. It is plausible that she devised methods to minimize this mark by adjusting the intensity or method of tourniquet application. Despite suspecting Munchausen syndrome, our decision to proceed with surgery was driven by concerns about potential complications of compartment syndrome. Throughout the entire process, despite our apprehensions regarding possible complications and the post-fasciotomy period, the patient remained remarkably calm and indifferent, consistent with their readiness to undergo significant surgical interventions, despite the knowledge that such procedures were unnecessary.

To best of our knowledge, there is only one reported Munchausen case in literature who resulted with fasciotomy [17]. Since tourniquet application is painful and disturbing process, this kind of self-mutilation might be less common among patients that seeks attention. In our case, while the diagnosis may be considered delayed regarding four possibly avoidable surgeries, the ultimate clarification of the issue without resulting in permanent neurovascular damage or morbidity can be considered as a partial success [18,19]. Given the potential complications of compartment syndrome, fasciotomies performed based on clinical findings appears warranted from a medical standpoint. We acknowledge that the extent of fasciotomy carried out during the second surgery and lack of intracompartmental pressure measurement are subjects to debate. It could be argued that, in cases of uncertainty, the forearm compartments should have also been opened. However, despite the limited time available before the operation, our preliminary diagnosis had been established, and the forearm was relatively undisturbed at that time. Therefore, we took an initiative and opted for less extensive approach for the second fasciotomy. The rapid reduction in swelling and paresthesia, along with complete resolution by the end of the follow-up, affirmed the adequacy of this decision.

## 4 Conclusion

It is of importance to consider Munchausen Syndrome as a potential cause, particularly in cases where clinical history and course of symptoms do not align with our experiences and cannot be reconciled with other possible diagnostic patterns. Surgeons should uphold surgical indications and principles without compromise, acknowledging that even in the cases of self-harm interventions may be necessary.

## Ethical approval

No ethical board approval required for single case report. Informed consent received from patient regarding permission to use presented data.

## Funding

No funding received for this report. There is no relationship with third parties.

## Author contribution

AV and AED wrote the manuscript, YY, CM, OT acquired the data, KA supervised the study.

## Guarantor

Dr. Arman Vahabi

## Consent

Written informed consent was obtained from the patient for publication and any accompanying images. A copy of the written consent is available for review by the Editor-in-Chief of this journal on request.

## Conflict of interest statement

Nothing to declare.
